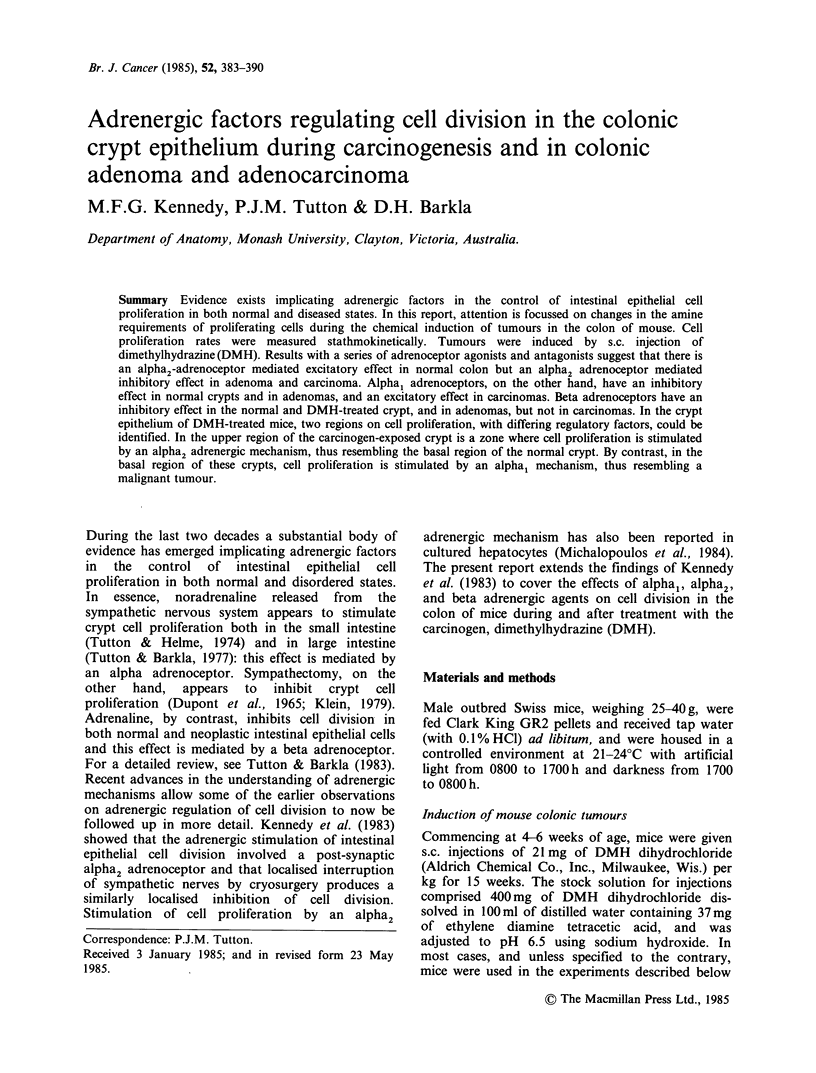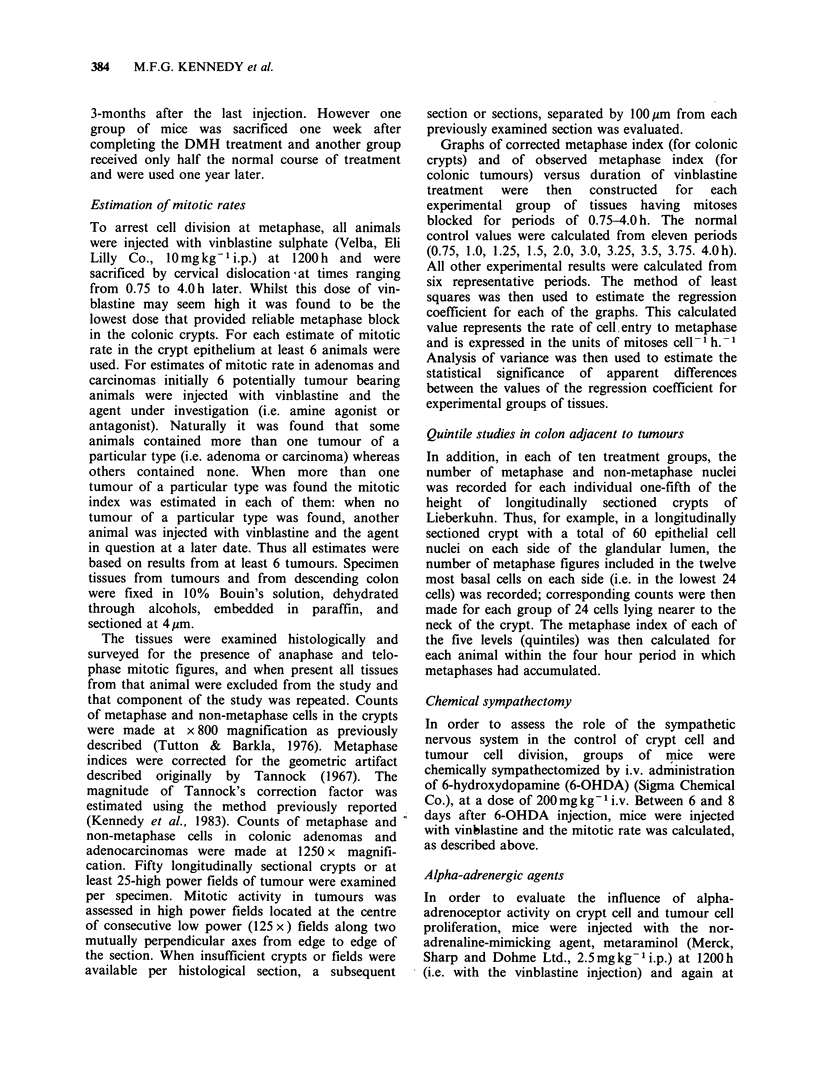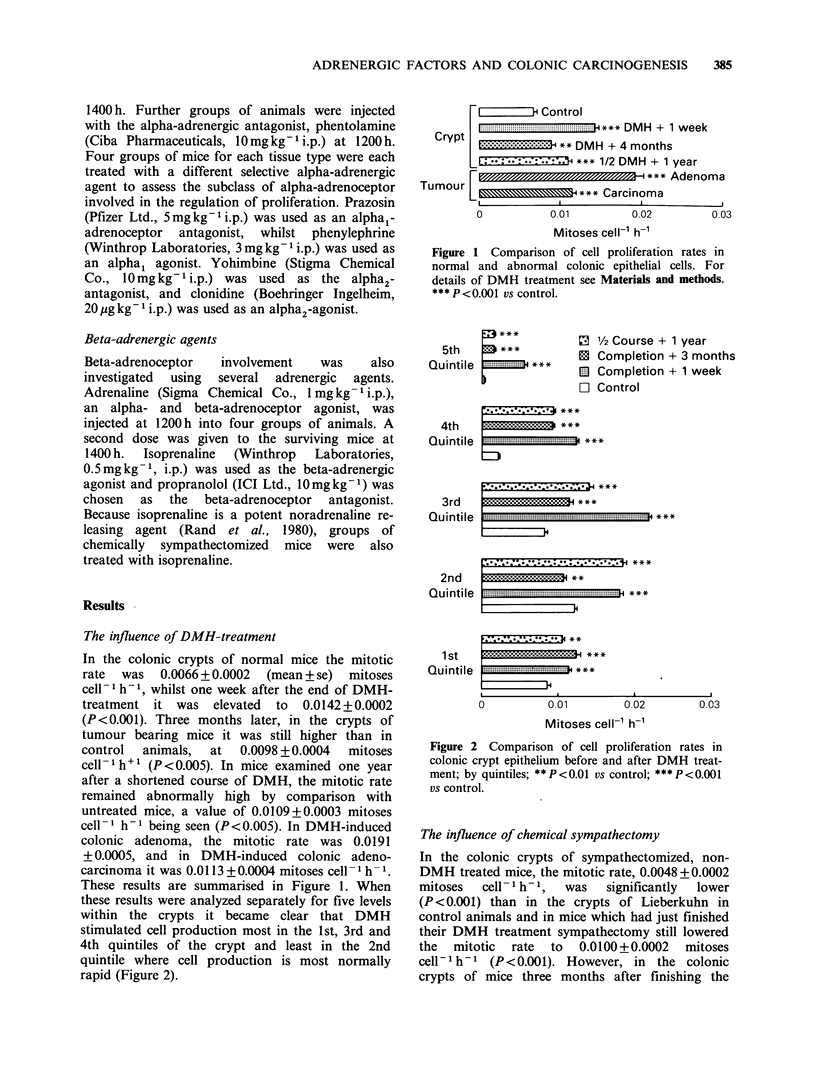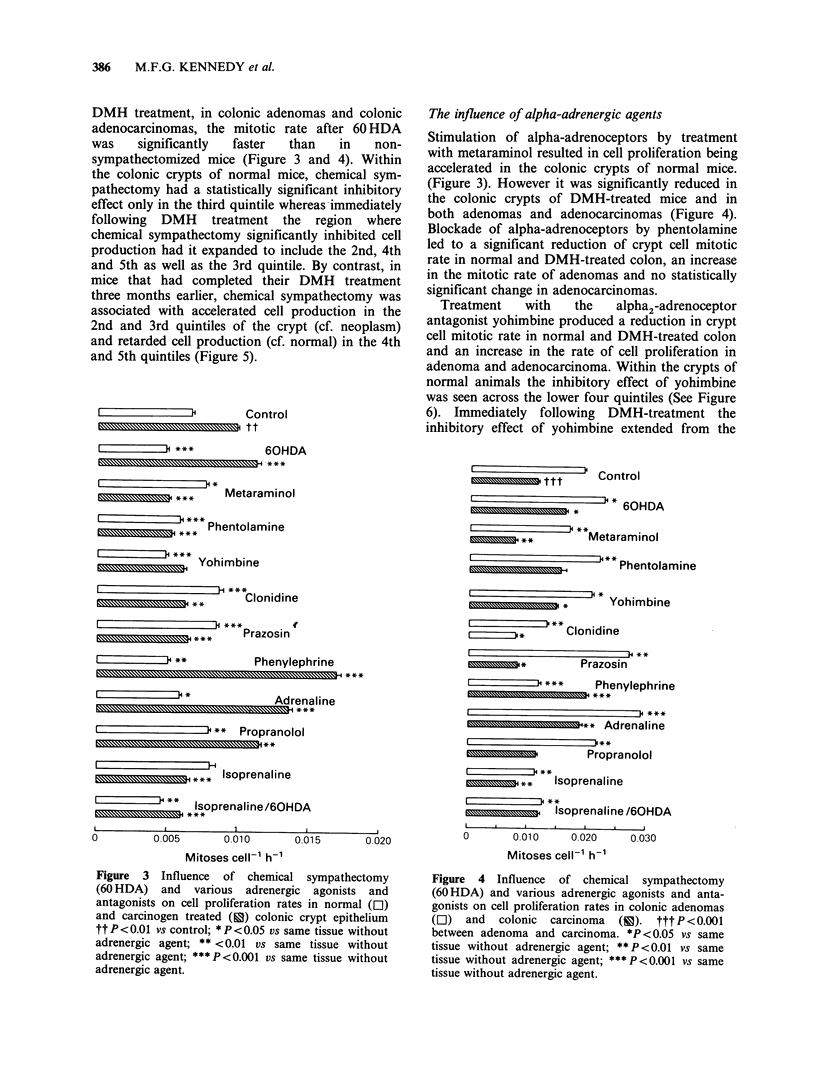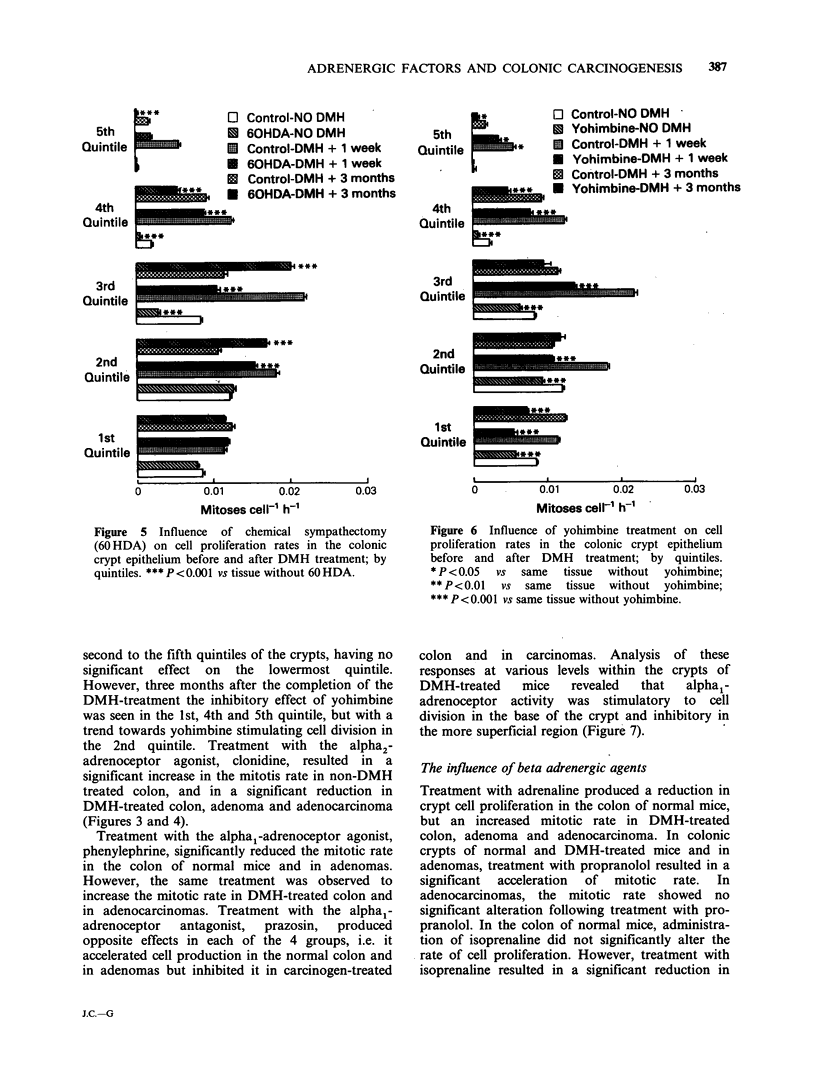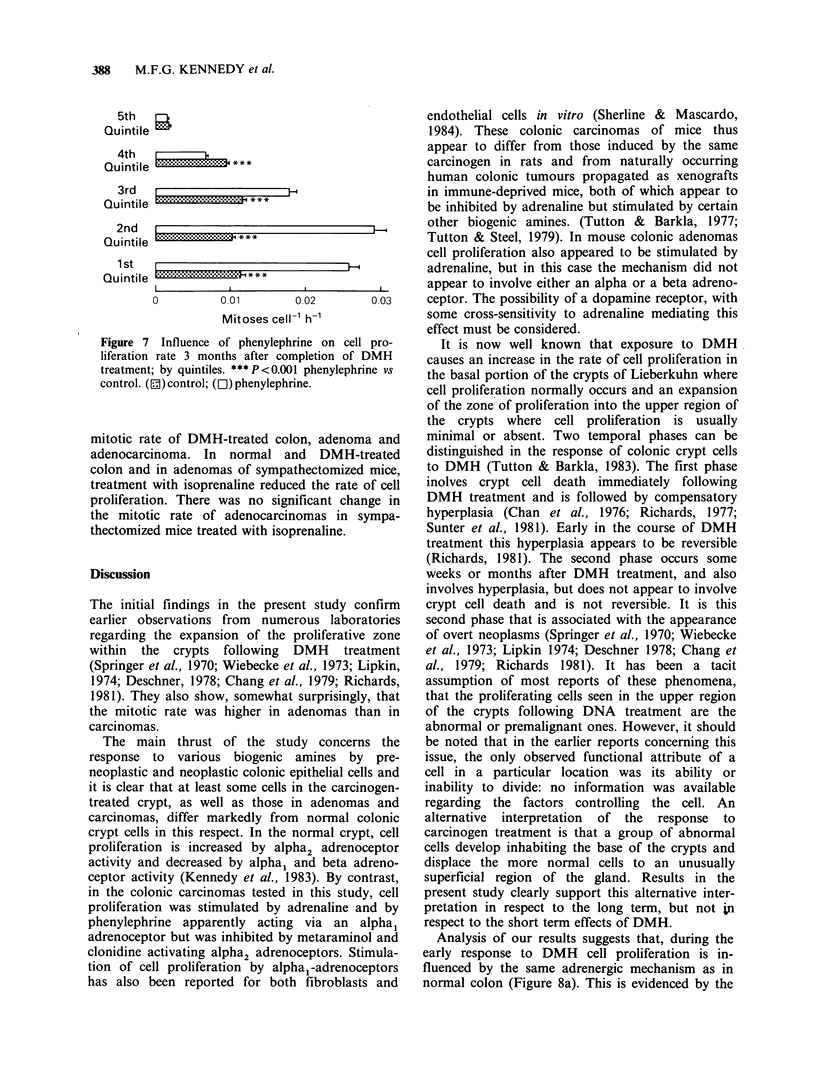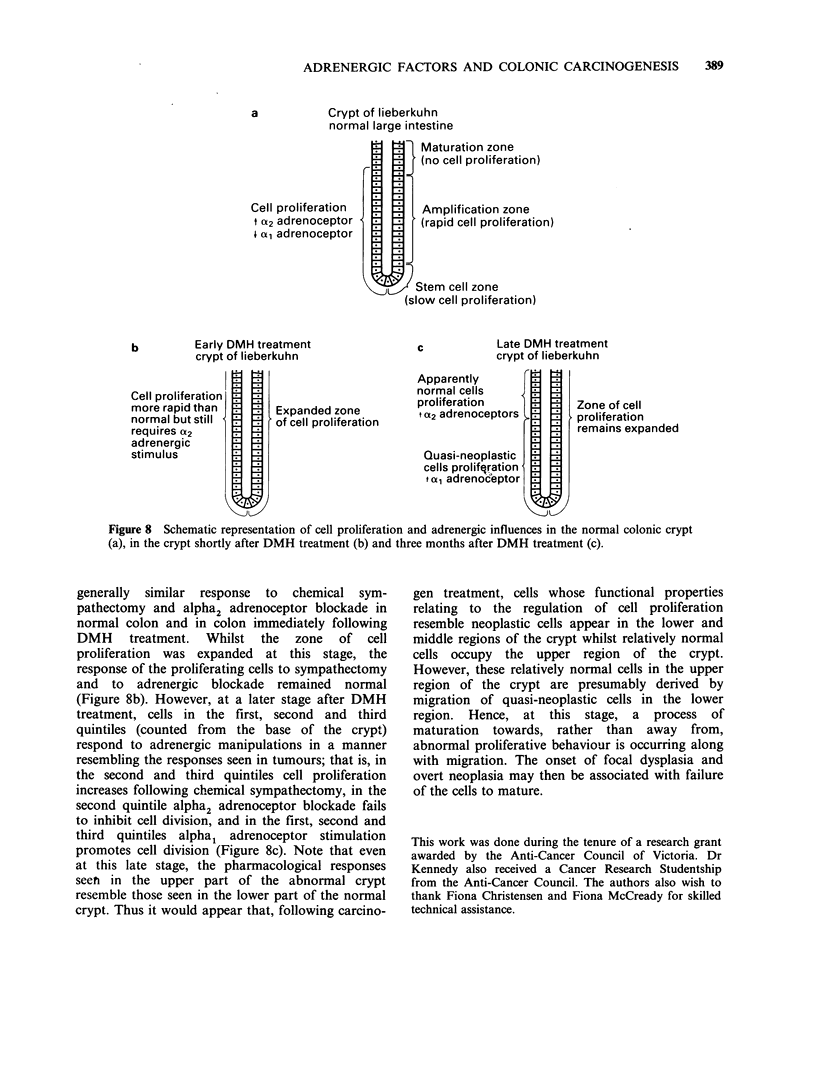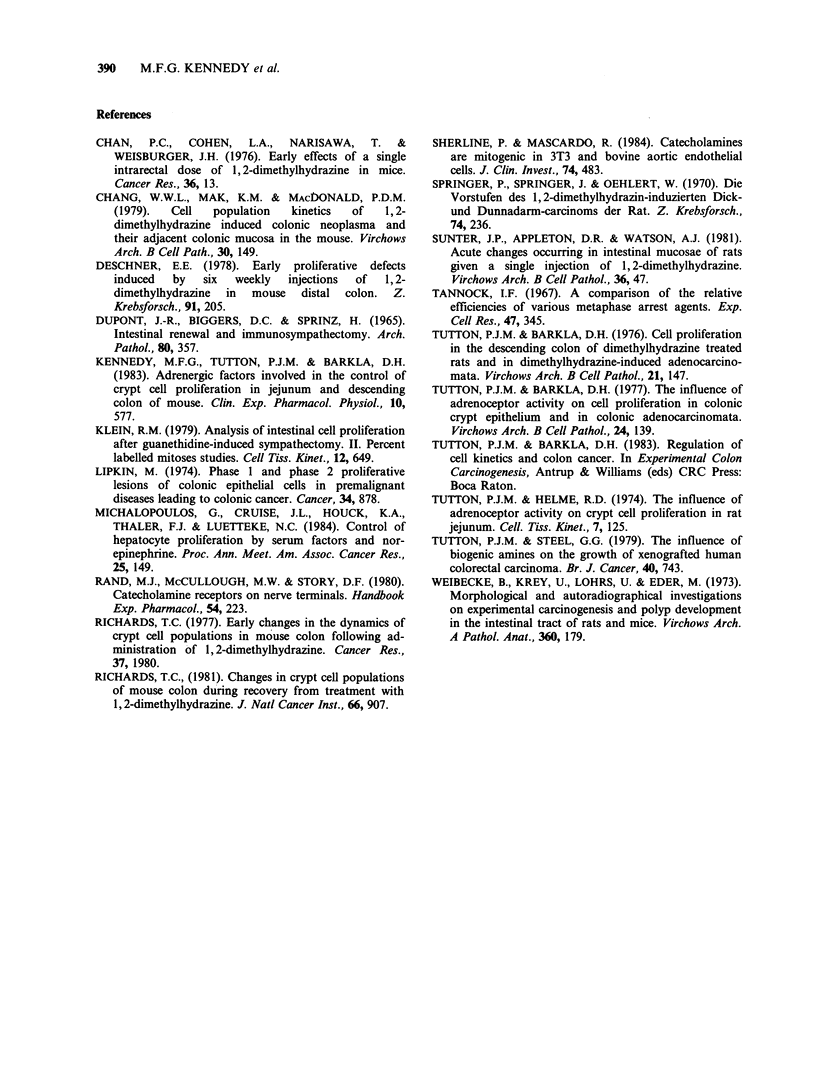# Adrenergic factors regulating cell division in the colonic crypt epithelium during carcinogenesis and in colonic adenoma and adenocarcinoma.

**DOI:** 10.1038/bjc.1985.205

**Published:** 1985-09

**Authors:** M. F. Kennedy, P. J. Tutton, D. H. Barkla

## Abstract

Evidence exists implicating adrenergic factors in the control of intestinal epithelial cell proliferation in both normal and diseased states. In this report, attention is focussed on changes in the amine requirements of proliferating cells during the chemical induction of tumours in the colon of mouse. Cell proliferation rates were measured stathmokinetically. Tumours were induced by s.c. injection of dimethylhydrazine (DMH). Results with a series of adrenoceptor agonists and antagonists suggest that there is an alpha 2-adrenoceptor mediated excitatory effect in normal colon but an alpha 2 adrenoceptor mediated inhibitory effect in adenoma and carcinoma. Alpha 1 adrenoceptors, on the other hand, have an inhibitory effect in normal crypts and in adenomas, and an excitatory effect in carcinomas. Beta adrenoceptors have an inhibitory effect in the normal and DMH-treated crypt, and in adenomas, but not in carcinomas. In the crypt epithelium of DMH-treated mice, two regions on cell proliferation, with differing regulatory factors, could be identified. In the upper region of the carcinogen-exposed crypt is a zone where cell proliferation is stimulated by an alpha 2 adrenergic mechanism, thus resembling the basal region of the normal crypt. By contrast, in the basal region of these crypts, cell proliferation is stimulated by an alpha 1 mechanism, thus resembling a malignant tumour.


*Br. J. Cancer* (1985), **52**, 383–390

### Adrenergic factors regulating cell division in the colonic crypt epithelium during carcinogenesis and in colonic adenoma and adenocarcinoma

M.F.G. Kennedy, P.J.M. Tutton & D.H. Barkla

*Department of Anatomy, Monash University, Clayton, Victoria, Australia.*

**Summary** Evidence exists implicating adrenergic factors in the control of intestinal epithelial cell proliferation in both normal and diseased states. In this report, attention is focussed on changes in the amine requirements of proliferating cells during the chemical induction of tumours in the colon of mouse. Cell proliferation rates were measured stathmokinetically. Tumours were induced by s.c. injection of dimethylhydrazine (DMH). Results with a series of adrenoceptor agonists and antagonists suggest that there is an alpha$_2$-adrenoceptor mediated excitatory effect in normal colon but an alpha$_2$ adrenoceptor mediated inhibitory effect in adenoma and carcinoma. Alpha$_1$ adrenoceptors, on the other hand, have an inhibitory effect in normal crypts and in adenomas, and an excitatory effect in carcinomas. Beta adrenoceptors have an inhibitory effect in the normal and DMH-treated crypt, and in adenomas, but not in carcinomas. In the crypt epithelium of DMH-treated mice, two regions on cell proliferation, with differing regulatory factors, could be identified. In the upper region of the carcinogen-exposed crypt is a zone where cell proliferation is stimulated by an alpha$_2$ adrenergic mechanism, thus resembling the basal region of the normal crypt. By contrast, in the basal region of these crypts, cell proliferation is stimulated by an alpha$_1$ mechanism, thus resembling a malignant tumour.

During the last two decades a substantial body of evidence has emerged implicating adrenergic factors in the control of intestinal epithelial cell proliferation in both normal and disordered states. In essence, noradrenaline released from the sympathetic nervous system appears to stimulate crypt cell proliferation both in the small intestine (Tutton & Helme, 1974) and in large intestine (Tutton & Barkla, 1977): this effect is mediated by an alpha adrenoceptor. Sympathectomy, on the other hand, appears to inhibit crypt cell proliferation (Dupont *et al.*, 1965; Klein, 1979). Adrenaline, by contrast, inhibits cell division in both normal and neoplastic intestinal epithelial cells and this effect is mediated by a beta adrenoceptor. For a detailed review, see Tutton & Barkla (1983). Recent advances in the understanding of adrenergic mechanisms allow some of the earlier observations on adrenergic regulation of cell division to now be followed up in more detail. Kennedy *et al.* (1983) showed that the adrenergic stimulation of intestinal epithelial cell division involved a post-synaptic alpha$_2$ adrenoceptor and that localised interruption of sympathetic nerves by cryosurgery produces a similarly localised inhibition of cell division. Stimulation of cell proliferation by an alpha$_2$ adrenergic mechanism has also been reported in cultured hepatocytes (Michalopoulos *et al.*, 1984). The present report extends the findings of Kennedy *et al.* (1983) to cover the effects of alpha$_1$, alpha$_2$, and beta adrenergic agents on cell division in the colon of mice during and after treatment with the carcinogen, dimethylhydrazine (DMH).

Correspondence: P.J.M. Tutton.

Received 3 January 1985; and in revised form 23 May 1985.

### Materials and methods

Male outbred Swiss mice, weighing 25–40 g, were fed Clark King GR2 pellets and received tap water (with 0.1% HCl) *ad libitum*, and were housed in a controlled environment at 21–24°C with artificial light from 0800 to 1700 h and darkness from 1700 to 0800 h.

### *Induction of mouse colonic tumours*

Commencing at 4–6 weeks of age, mice were given s.c. injections of 21 mg of DMH dihydrochloride (Aldrich Chemical Co., Inc., Milwaukee, Wis.) per kg for 15 weeks. The stock solution for injections comprised 400 mg of DMH dihydrochloride dissolved in 100 ml of distilled water containing 37 mg of ethylene diamine tetracetic acid, and was adjusted to pH 6.5 using sodium hydroxide. In most cases, and unless specified to the contrary, mice were used in the experiments described below

© The Macmillan Press Ltd., 1985

3-months after the last injection. However one group of mice was sacrificed one week after completing the DMH treatment and another group received only half the normal course of treatment and were used one year later.

*Estimation of mitotic rates*

To arrest cell division at metaphase, all animals were injected with vinblastine sulphate (Velba, Eli Lilly Co., $10\,mg\,kg^{-1}$ i.p.) at 1200 h and were sacrificed by cervical dislocation at times ranging from 0.75 to 4.0 h later. Whilst this dose of vinblastine may seem high it was found to be the lowest dose that provided reliable metaphase block in the colonic crypts. For each estimate of mitotic rate in the crypt epithelium at least 6 animals were used. For estimates of mitotic rate in adenomas and carcinomas initially 6 potentially tumour bearing animals were injected with vinblastine and the agent under investigation (i.e. amine agonist or antagonist). Naturally it was found that some animals contained more than one tumour of a particular type (i.e. adenoma or carcinoma) whereas others contained none. When more than one tumour of a particular type was found the mitotic index was estimated in each of them: when no tumour of a particular type was found, another animal was injected with vinblastine and the agent in question at a later date. Thus all estimates were based on results from at least 6 tumours. Specimen tissues from tumours and from descending colon were fixed in 10% Bouin's solution, dehydrated through alcohols, embedded in paraffin, and sectioned at $4\,\mu m$.

The tissues were examined histologically and surveyed for the presence of anaphase and telophase mitotic figures, and when present all tissues from that animal were excluded from the study and that component of the study was repeated. Counts of metaphase and non-metaphase cells in the crypts were made at $\times 800$ magnification as previously described (Tutton & Barkla, 1976). Metaphase indices were corrected for the geometric artifact described originally by Tannock (1967). The magnitude of Tannock's correction factor was estimated using the method previously reported (Kennedy *et al.*, 1983). Counts of metaphase and non-metaphase cells in colonic adenomas and adenocarcinomas were made at $1250\times$ magnification. Fifty longitudinally sectional crypts or at least 25-high power fields of tumour were examined per specimen. Mitotic activity in tumours was assessed in high power fields located at the centre of consecutive low power ($125\times$) fields along two mutually perpendicular axes from edge to edge of the section. When insufficient crypts or fields were available per histological section, a subsequent section or sections, separated by $100\,\mu m$ from each previously examined section was evaluated.

Graphs of corrected metaphase index (for colonic crypts) and of observed metaphase index (for colonic tumours) versus duration of vinblastine treatment were then constructed for each experimental group of tissues having mitoses blocked for periods of 0.75–4.0 h. The normal control values were calculated from eleven periods (0.75, 1.0, 1.25, 1.5, 2.0, 3.0, 3.25, 3.5, 3.75. 4.0 h). All other experimental results were calculated from six representative periods. The method of least squares was then used to estimate the regression coefficient for each of the graphs. This calculated value represents the rate of cell entry to metaphase and is expressed in the units of mitoses $cell^{-1}\,h.^{-1}$ Analysis of variance was then used to estimate the statistical significance of apparent differences between the values of the regression coefficient for experimental groups of tissues.

*Quintile studies in colon adjacent to tumours*

In addition, in each of ten treatment groups, the number of metaphase and non-metaphase nuclei was recorded for each individual one-fifth of the height of longitudinally sectioned crypts of Lieberkuhn. Thus, for example, in a longitudinally sectioned crypt with a total of 60 epithelial cell nuclei on each side of the glandular lumen, the number of metaphase figures included in the twelve most basal cells on each side (i.e. in the lowest 24 cells) was recorded; corresponding counts were then made for each group of 24 cells lying nearer to the neck of the crypt. The metaphase index of each of the five levels (quintiles) was then calculated for each animal within the four hour period in which metaphases had accumulated.

*Chemical sympathectomy*

In order to assess the role of the sympathetic nervous system in the control of crypt cell and tumour cell division, groups of mice were chemically sympathectomized by i.v. administration of 6-hydroxydopamine (6-OHDA) (Sigma Chemical Co.), at a dose of $200\,mg\,kg^{-1}$ i.v. Between 6 and 8 days after 6-OHDA injection, mice were injected with vinblastine and the mitotic rate was calculated, as described above.

*Alpha-adrenergic agents*

In order to evaluate the influence of alpha-adrenoceptor activity on crypt cell and tumour cell proliferation, mice were injected with the noradrenaline-mimicking agent, metaraminol (Merck, Sharp and Dohme Ltd., $2.5\,mg\,kg^{-1}$ i.p.) at 1200 h (i.e. with the vinblastine injection) and again at

1400 h. Further groups of animals were injected with the alpha-adrenergic antagonist, phentolamine (Ciba Pharmaceuticals, 10 mg kg$^{-1}$ i.p.) at 1200 h. Four groups of mice for each tissue type were each treated with a different selective alpha-adrenergic agent to assess the subclass of alpha-adrenoceptor involved in the regulation of proliferation. Prazosin (Pfizer Ltd., 5 mg kg$^{-1}$ i.p.) was used as an alpha$_1$-adrenoceptor antagonist, whilst phenylephrine (Winthrop Laboratories, 3 mg kg$^{-1}$ i.p.) was used as an alpha$_1$ agonist. Yohimbine (Stigma Chemical Co., 10 mg kg$^{-1}$ i.p.) was used as the alpha$_2$-antagonist, and clonidine (Boehringer Ingelheim, 20 $\mu$g kg$^{-1}$ i.p.) was used as an alpha$_2$-agonist.

*Beta-adrenergic agents*

Beta-adrenoceptor involvement was also investigated using several adrenergic agents. Adrenaline (Sigma Chemical Co., 1 mg kg$^{-1}$ i.p.), an alpha- and beta-adrenoceptor agonist, was injected at 1200 h into four groups of animals. A second dose was given to the surviving mice at 1400 h. Isoprenaline (Winthrop Laboratories, 0.5 mg kg$^{-1}$, i.p.) was used as the beta-adrenergic agonist and propranolol (ICI Ltd., 10 mg kg$^{-1}$) was chosen as the beta-adrenoceptor antagonist. Because isoprenaline is a potent noradrenaline releasing agent (Rand *et al.*, 1980), groups of chemically sympathectomized mice were also treated with isoprenaline.

### Results

*The influence of DMH-treatment*

In the colonic crypts of normal mice the mitotic rate was 0.0066 ± 0.0002 (mean ± se) mitoses cell$^{-1}$ h$^{-1}$, whilst one week after the end of DMH-treatment it was elevated to 0.0142 ± 0.0002 ($P<0.001$). Three months later, in the crypts of tumour bearing mice it was still higher than in control animals, at 0.0098 ± 0.0004 mitoses cell$^{-1}$ h$^{+1}$ ($P<0.005$). In mice examined one year after a shortened course of DMH, the mitotic rate remained abnormally high by comparison with untreated mice, a value of 0.0109 ± 0.0003 mitoses cell$^{-1}$ h$^{-1}$ being seen ($P<0.005$). In DMH-induced colonic adenoma, the mitotic rate was 0.0191 ± 0.0005, and in DMH-induced colonic adenocarcinoma it was 0.0113 ± 0.0004 mitoses cell$^{-1}$ h$^{-1}$. These results are summarised in Figure 1. When these results were analyzed separately for five levels within the crypts it became clear that DMH stimulated cell production most in the 1st, 3rd and 4th quintiles of the crypt and least in the 2nd quintile where cell production is most normally rapid (Figure 2).

**Figure 1** Comparison of cell proliferation rates in normal and abnormal colonic epithelial cells. For details of DMH treatment see **Materials and methods**. *** $P<0.001$ *vs* control.

**Figure 2** Comparison of cell proliferation rates in colonic crypt epithelium before and after DMH treatment; by quintiles; ** $P<0.01$ *vs* control; *** $P<0.001$ *vs* control.

*The influence of chemical sympathectomy*

In the colonic crypts of sympathectomized, non-DMH treated mice, the mitotic rate, 0.0048 ± 0.0002 mitoses cell$^{-1}$ h$^{-1}$, was significantly lower ($P<0.001$) than in the crypts of Lieberkuhn in control animals and in mice which had just finished their DMH treatment sympathectomy still lowered the mitotic rate to 0.0100 ± 0.0002 mitoses cell$^{-1}$ h$^{-1}$ ($P<0.001$). However, in the colonic crypts of mice three months after finishing the

DMH treatment, in colonic adenomas and colonic adenocarcinomas, the mitotic rate after 60 HDA was significantly faster than in non-sympathectomized mice (Figure 3 and 4). Within the colonic crypts of normal mice, chemical sympathectomy had a statistically significant inhibitory effect only in the third quintile whereas immediately following DMH treatment the region where chemical sympathectomy significantly inhibited cell production had it expanded to include the 2nd, 4th and 5th as well as the 3rd quintile. By contrast, in mice that had completed their DMH treatment three months earlier, chemical sympathectomy was associated with accelerated cell production in the 2nd and 3rd quintiles of the crypt (cf. neoplasm) and retarded cell production (cf. normal) in the 4th and 5th quintiles (Figure 5).

*The influence of alpha-adrenergic agents*

Stimulation of alpha-adrenoceptors by treatment with metaraminol resulted in cell proliferation being accelerated in the colonic crypts of normal mice. (Figure 3). However it was significantly reduced in the colonic crypts of DMH-treated mice and in both adenomas and adenocarcinomas (Figure 4). Blockade of alpha-adrenoceptors by phentolamine led to a significant reduction of crypt cell mitotic rate in normal and DMH-treated colon, an increase in the mitotic rate of adenomas and no statistically significant change in adenocarcinomas.

Treatment with the $alpha_2$-adrenoceptor antagonist yohimbine produced a reduction in crypt cell mitotic rate in normal and DMH-treated colon and an increase in the rate of cell proliferation in adenoma and adenocarcinoma. Within the crypts of normal animals the inhibitory effect of yohimbine was seen across the lower four quintiles (See Figure 6). Immediately following DMH-treatment the inhibitory effect of yohimbine extended from the

**Figure 3** Influence of chemical sympathectomy (60 HDA) and various adrenergic agonists and antagonists on cell proliferation rates in normal (□) and carcinogen treated (▧) colonic crypt epithelium †† $P<0.01$ *vs* control; * $P<0.05$ *vs* same tissue without adrenergic agent; ** $<0.01$ *vs* same tissue without adrenergic agent; *** $P<0.001$ *vs* same tissue without adrenergic agent.

**Figure 4** Influence of chemical sympathectomy (60 HDA) and various adrenergic agonists and antagonists on cell proliferation rates in colonic adenomas (□) and colonic carcinoma (▧). ††† $P<0.001$ between adenoma and carcinoma. * $P<0.05$ *vs* same tissue without adrenergic agent; ** $P<0.01$ *vs* same tissue without adrenergic agent; *** $P<0.001$ *vs* same tissue without adrenergic agent.

**Figure 5** Influence of chemical sympathectomy (60 HDA) on cell proliferation rates in the colonic crypt epithelium before and after DMH treatment; by quintiles. *** $P<0.001$ *vs* tissue without 60 HDA.

**Figure 6** Influence of yohimbine treatment on cell proliferation rates in the colonic crypt epithelium before and after DMH treatment; by quintiles. * $P<0.05$ *vs* same tissue without yohimbine; ** $P<0.01$ *vs* same tissue without yohimbine; *** $P<0.001$ *vs* same tissue without yohimbine.

second to the fifth quintiles of the crypts, having no significant effect on the lowermost quintile. However, three months after the completion of the DMH-treatment the inhibitory effect of yohimbine was seen in the 1st, 4th and 5th quintile, but with a trend towards yohimbine stimulating cell division in the 2nd quintile. Treatment with the alpha$_2$-adrenoceptor agonist, clonidine, resulted in a significant increase in the mitotis rate in non-DMH treated colon, and in a significant reduction in DMH-treated colon, adenoma and adenocarcinoma (Figures 3 and 4).

Treatment with the alpha$_1$-adrenoceptor agonist, phenylephrine, significantly reduced the mitotic rate in the colon of normal mice and in adenomas. However, the same treatment was observed to increase the mitotic rate in DMH-treated colon and in adenocarcinomas. Treatment with the alpha$_1$-adrenoceptor antagonist, prazosin, produced opposite effects in each of the 4 groups, i.e. it accelerated cell production in the normal colon and in adenomas but inhibited it in carcinogen-treated colon and in carcinomas. Analysis of these responses at various levels within the crypts of DMH-treated mice revealed that alpha$_1$-adrenoceptor activity was stimulatory to cell division in the base of the crypt and inhibitory in the more superficial region (Figure 7).

### *The influence of beta adrenergic agents*

Treatment with adrenaline produced a reduction in crypt cell proliferation in the colon of normal mice, but an increased mitotic rate in DMH-treated colon, adenoma and adenocarcinoma. In colonic crypts of normal and DMH-treated mice and in adenomas, treatment with propranolol resulted in a significant acceleration of mitotic rate. In adenocarcinomas, the mitotic rate showed no significant alteration following treatment with propranolol. In the colon of normal mice, administration of isoprenaline did not significantly alter the rate of cell proliferation. However, treatment with isoprenaline resulted in a significant reduction in

**Figure 7** Influence of phenylephrine on cell proliferation rate 3 months after completion of DMH treatment; by quintiles. *** $P<0.001$ phenylephrine *vs* control. (▩) control; (□) phenylephrine.

mitotic rate of DMH-treated colon, adenoma and adenocarcinoma. In normal and DMH-treated colon and in adenomas of sympathectomized mice, treatment with isoprenaline reduced the rate of cell proliferation. There was no significant change in the mitotic rate of adenocarcinomas in sympathectomized mice treated with isoprenaline.

### Discussion

The initial findings in the present study confirm earlier observations from numerous laboratories regarding the expansion of the proliferative zone within the crypts following DMH treatment (Springer *et al.*, 1970; Wiebecke *et al.*, 1973; Lipkin, 1974; Deschner, 1978; Chang *et al.*, 1979; Richards, 1981). They also show, somewhat surprisingly, that the mitotic rate was higher in adenomas than in carcinomas.

The main thrust of the study concerns the response to various biogenic amines by preneoplastic and neoplastic colonic epithelial cells and it is clear that at least some cells in the carcinogen-treated crypt, as well as those in adenomas and carcinomas, differ markedly from normal colonic crypt cells in this respect. In the normal crypt, cell proliferation is increased by $alpha_2$ adrenoceptor activity and decreased by $alpha_1$ and beta adrenoceptor activity (Kennedy *et al.*, 1983). By contrast, in the colonic carcinomas tested in this study, cell proliferation was stimulated by adrenaline and by phenylephrine apparently acting via an $alpha_1$ adrenoceptor but was inhibited by metaraminol and clonidine activating $alpha_2$ adrenoceptors. Stimulation of cell proliferation by $alpha_1$-adrenoceptors has also been reported for both fibroblasts and endothelial cells *in vitro* (Sherline & Mascardo, 1984). These colonic carcinomas of mice thus appear to differ from those induced by the same carcinogen in rats and from naturally occurring human colonic tumours propagated as xenografts in immune-deprived mice, both of which appear to be inhibited by adrenaline but stimulated by certain other biogenic amines. (Tutton & Barkla, 1977; Tutton & Steel, 1979). In mouse colonic adenomas cell proliferation also appeared to be stimulated by adrenaline, but in this case the mechanism did not appear to involve either an alpha or a beta adrenoceptor. The possibility of a dopamine receptor, with some cross-sensitivity to adrenaline mediating this effect must be considered.

It is now well known that exposure to DMH causes an increase in the rate of cell proliferation in the basal portion of the crypts of Lieberkuhn where cell proliferation normally occurs and an expansion of the zone of proliferation into the upper region of the crypts where cell proliferation is usually minimal or absent. Two temporal phases can be distinguished in the response of colonic crypt cells to DMH (Tutton & Barkla, 1983). The first phase inolves crypt cell death immediately following DMH treatment and is followed by compensatory hyperplasia (Chan *et al.*, 1976; Richards, 1977; Sunter *et al.*, 1981). Early in the course of DMH treatment this hyperplasia appears to be reversible (Richards, 1981). The second phase occurs some weeks or months after DMH treatment, and also involves hyperplasia, but does not appear to involve crypt cell death and is not reversible. It is this second phase that is associated with the appearance of overt neoplasms (Springer *et al.*, 1970; Wiebecke *et al.*, 1973; Lipkin 1974; Deschner 1978; Chang *et al.*, 1979; Richards 1981). It has been a tacit assumption of most reports of these phenomena, that the proliferating cells seen in the upper region of the crypts following DNA treatment are the abnormal or premalignant ones. However, it should be noted that in the earlier reports concerning this issue, the only observed functional attribute of a cell in a particular location was its ability or inability to divide: no information was available regarding the factors controlling the cell. An alternative interpretation of the response to carcinogen treatment is that a group of abnormal cells develop inhabiting the base of the crypts and displace the more normal cells to an unusually superficial region of the gland. Results in the present study clearly support this alternative interpretation in respect to the long term, but not in respect to the short term effects of DMH.

Analysis of our results suggests that, during the early response to DMH cell proliferation is influenced by the same adrenergic mechanism as in normal colon (Figure 8a). This is evidenced by the

**Figure 8** Schematic representation of cell proliferation and adrenergic influences in the normal colonic crypt (a), in the crypt shortly after DMH treatment (b) and three months after DMH treatment (c).

generally similar response to chemical sympathectomy and $alpha_2$ adrenoceptor blockade in normal colon and in colon immediately following DMH treatment. Whilst the zone of cell proliferation was expanded at this stage, the response of the proliferating cells to sympathectomy and to adrenergic blockade remained normal (Figure 8b). However, at a later stage after DMH treatment, cells in the first, second and third quintiles (counted from the base of the crypt) respond to adrenergic manipulations in a manner resembling the responses seen in tumours; that is, in the second and third quintiles cell proliferation increases following chemical sympathectomy, in the second quintile $alpha_2$ adrenoceptor blockade fails to inhibit cell division, and in the first, second and third quintiles $alpha_1$ adrenoceptor stimulation promotes cell division (Figure 8c). Note that even at this late stage, the pharmacological responses seen in the upper part of the abnormal crypt resemble those seen in the lower part of the normal crypt. Thus it would appear that, following carcinogen treatment, cells whose functional properties relating to the regulation of cell proliferation resemble neoplastic cells appear in the lower and middle regions of the crypt whilst relatively normal cells occupy the upper region of the crypt. However, these relatively normal cells in the upper region of the crypt are presumably derived by migration of quasi-neoplastic cells in the lower region. Hence, at this stage, a process of maturation towards, rather than away from, abnormal proliferative behaviour is occurring along with migration. The onset of focal dysplasia and overt neoplasia may then be associated with failure of the cells to mature.

This work was done during the tenure of a research grant awarded by the Anti-Cancer Council of Victoria. Dr Kennedy also received a Cancer Research Studentship from the Anti-Cancer Council. The authors also wish to thank Fiona Christensen and Fiona McCready for skilled technical assistance.

### References

CHAN, P.C., COHEN, L.A., NARISAWA, T. & WEISBURGER, J.H. (1976). Early effects of a single intrarectal dose of 1,2-dimethylhydrazine in mice. *Cancer Res.*, **36,** 13.

CHANG, W.W.L., MAK, K.M. & MACDONALD, P.D.M. (1979). Cell population kinetics of 1,2-dimethylhydrazine induced colonic neoplasma and their adjacent colonic mucosa in the mouse. *Virchows Arch. B Cell Path.*, **30,** 149.

DESCHNER, E.E. (1978). Early proliferative defects induced by six weekly injections of 1,2-dimethylhydrazine in mouse distal colon. *Z. Krebsforsch.*, **91,** 205.

DUPONT, J.-R., BIGGERS, D.C. & SPRINZ, H. (1965). Intestinal renewal and immunosympathectomy. *Arch. Pathol.*, **80,** 357.

KENNEDY, M.F.G., TUTTON, P.J.M. & BARKLA, D.H. (1983). Adrenergic factors involved in the control of crypt cell proliferation in jejunum and descending colon of mouse. *Clin. Exp. Pharmacol. Physiol.*, **10,** 577.

KLEIN, R.M. (1979). Analysis of intestinal cell proliferation after guanethidine-induced sympathectomy. II. Percent labelled mitoses studies. *Cell Tiss. Kinet.*, **12,** 649.

LIPKIN, M. (1974). Phase 1 and phase 2 proliferative lesions of colonic epithelial cells in premalignant diseases leading to colonic cancer. *Cancer*, **34,** 878.

MICHALOPOULOS, G., CRUISE, J.L., HOUCK, K.A., THALER, F.J. & LUETTEKE, N.C. (1984). Control of hepatocyte proliferation by serum factors and norepinephrine. *Proc. Ann. Meet. Am. Assoc. Cancer Res.*, **25,** 149.

RAND, M.J., McCULLOUGH, M.W. & STORY, D.F. (1980). Catecholamine receptors on nerve terminals. *Handbook Exp. Pharmacol.*, **54,** 223.

RICHARDS, T.C. (1977). Early changes in the dynamics of crypt cell populations in mouse colon following administration of 1,2-dimethylhydrazine. *Cancer Res.*, **37,** 1980.

RICHARDS, T.C., (1981). Changes in crypt cell populations of mouse colon during recovery from treatment with 1,2-dimethylhydrazine. *J. Natl Cancer Inst.*, **66,** 907.

SHERLINE, P. & MASCARDO, R. (1984). Catecholamines are mitogenic in 3T3 and bovine aortic endothelial cells. *J. Clin. Invest.*, **74,** 483.

SPRINGER, P., SPRINGER, J. & OEHLERT, W. (1970). Die Vorstufen des 1,2-dimethylhydrazin-induzierten Dick- und Dunnadarm-carcinoms der Rat. *Z. Krebsforsch.*, **74,** 236.

SUNTER, J.P., APPLETON, D.R. & WATSON, A.J. (1981). Acute changes occurring in intestinal mucosae of rats given a single injection of 1,2-dimethylhydrazine. *Virchows Arch. B Cell Pathol.*, **36,** 47.

TANNOCK, I.F. (1967). A comparison of the relative efficiencies of various metaphase arrest agents. *Exp. Cell Res.*, **47,** 345.

TUTTON, P.J.M. & BARKLA, D.H. (1976). Cell proliferation in the descending colon of dimethylhydrazine treated rats and in dimethylhydrazine-induced adenocarcinomata. *Virchows Arch. B Cell Pathol.*, **21,** 147.

TUTTON, P.J.M. & BARKLA, D.H. (1977). The influence of adrenoceptor activity on cell proliferation in colonic crypt epithelium and in colonic adenocarcinomata. *Virchows Arch. B Cell Pathol.*, **24,** 139.

TUTTON, P.J.M. & BARKLA, D.H. (1983). Regulation of cell kinetics and colon cancer. In *Experimental Colon Carcinogenesis*, Antrup & Williams (eds) CRC Press: Boca Raton.

TUTTON, P.J.M. & HELME, R.D. (1974). The influence of adrenoceptor activity on crypt cell proliferation in rat jejunum. *Cell. Tiss. Kinet.*, **7,** 125.

TUTTON, P.J.M. & STEEL, G.G. (1979). The influence of biogenic amines on the growth of xenografted human colorectal carcinoma. *Br. J. Cancer*, **40,** 743.

WEIBECKE, B., KREY, U., LOHRS, U. & EDER, M. (1973). Morphological and autoradiographical investigations on experimental carcinogenesis and polyp development in the intestinal tract of rats and mice. *Virchows Arch. A Pathol. Anat.*, **360,** 179.